# Nine types of recommendations, guidelines and policies: an exploratory test of a proposed typology of physical activity promotion documents

**DOI:** 10.1186/s13690-019-0381-x

**Published:** 2019-12-02

**Authors:** Peter Gelius, Sven Messing, Karim Abu-Omar

**Affiliations:** FAU Erlangen-Nürnberg, Department of Sport Science and Sport, Gebbertstraße 123b, 91058 Erlangen, Germany

**Keywords:** Public health, Health promotion, Physical activity, Documents, Typology, Recommendation, Guideline, Policy

## Abstract

**Background:**

The field of physical activity abounds with recommendations, guidelines, action plans and other documents published by experts, organizations and institutions at the national and international level. However, working with these documents is difficult since similar names (e.g. “recommendations”) may be used to label substantially different contents, while identical topics may hide behind different monikers (e.g. “guidelines” and “strategy”).

**Methods:**

We built on an existing framework conceptualizing categories of physical activity evidence and on the Doern continuum for policy instruments to develop a nine-field matrix that classifies physical activity-related publications based on their evidence type and degree of coercion. We used a selection of eleven physical activity documents to perform an exploratory test of the functions and utility of the typology.

**Results:**

Placing central physical activity documents into the typology shows that recommendations, guidelines, and policies are found across the entire matrix, regardless of their denomination. It also suggests that some documents transcend boundaries between types by falling into more than one category, and that some categories may be underrepresented in current physical activity promotion.

**Conclusions:**

A typology to classify physical activity guidelines, recommendations, and policies can help us acquire a better overview of the landscape of existing physical activity documents than simple distinctions based on document names. It may guide both current initiatives and future development work in the field. It could also serve as a point of departure for future research, as conducting systematic overviews of the literature based on this typology may help reveal important gaps in current physical activity promotion.

## Background

Considering the abundance of documents being issued on the subject, one might easily conclude that we are living in the golden age of physical activity promotion. Labeled as recommendations, guidelines, good practice documents, policies, strategies, action plans, and calls for action by various actors at both national and international level, these documents have defined how much physical activity one should do [1, 2], what type of interventions organizations and governments should pursue to promote physical activity [3], or even which public policies should be implemented to promote physical activity at the population level [4].

However, making sense of these documents is seriously hampered by the fact that it is often difficult to discern their perspective, thrust, target groups, and political relevance. For example, documents with similar names (e.g. the WHO Global *Recommendations* on Physical Activity for Health [5] and the EU Council *Recommendation* on promoting Health-Enhancing Physical Activity across Sectors [4], emphases added) may deal with entirely different classes of behavior (in this case, individual physical activity and policy action on physical activity, respectively). Conversely, papers may be very similar in nature, but their names may suggest entirely different classes of publications (e.g. the EU Council *Recommendation* on promoting Health-Enhancing Physical Activity across Sectors [4] and the WHO Physical Activity *Strategy* for the European Region [6], emphases added). In addition, the potential reach and impact of the various “recommendations” related to physical activity may vary significantly depending on who published them. All of this has consequences for how we should use these documents, which of them we should pay particular attention to, and which of them we should refer to for different purposes of research, health promotion advocacy, and program design.

To our best of knowledge, there is currently no framework to improve our understanding of the different classes of physical activity promotion documents. This article attempts to address this gap by proposing a theory-based typology and by conducting an exploratory test of its utility using a set of select documents.

## Methods

In a first step, we developed a typology of documents based on two dimensions: “category of physical activity evidence” and “degree of coercion”. The first dimension pays tribute to the fact that existing policy documents relate to different areas of physical activity promotion. In a scoping review attempting to capture the full “extent, range and nature” of physical activity research, Rütten et al. [7] found that one can distinguish between three categories of scientific findings, each of which is fed by a distinctive strand and tradition of research: evidence on the relationship between physical activity and individual health (category I), evidence on interventions to promote physical activity (category II), and evidence on organizational (e.g. school-based) and public (i.e. governmental) physical activity policy (category III). We suggest that most existing recommendations, guidelines, strategies and action plans can either be filed into one of these three categories or placed at the boundary between two of them.

The second dimension is borrowed from political science, specifically from research on policy instruments [8, 9]. Within this body of literature, important theoretical concepts to classify the different tools available to governments have traditionally been their amount of “regulation” [10] or the “likelihood of sanctions” [11, 12] that they imply. Doern and his colleagues further developed these ideas into a continuum of policy instruments with different “degrees of legitimate coercion” [13], ranging from self-regulation via soft measures such as exhortation to highly coercive instruments such as direct regulation.

We propose to transfer this logic to physical activity promotion and to apply it to structure the abundance of available documents. This proposal is based on the observation that documents come from actors with substantially varying degrees of political influence and power, and that their degree of coercion will naturally vary depending on their origin. For example, a recommendation issued by a group of researchers may be widely received and influence further research and policy-making, but it is less binding for national policy-making than one issued by a national ministry. Inter- and supranational organizations take an intermediate position on this continuum: By virtue of its own constitution (Article 2) [14], the role of WHO is limited to providing assistance to Member States; it cannot prescribe any specific policies or measures to be taken by national governments. Similarly, the Treaty on the Functioning of the European Union [15] stipulates that the EU only has the competence to “support, coordinate or supplement the actions of the Member States” (Article 6) in the fields most relevant to physical activity promotion, esp. health and sport but also education and tourism. Consequently, although documents on physical activity promotion issued by WHO and the EU receive widespread attention, one has to assume that they are less compulsive than those published by national governments. It is important to note that this situation is somewhat specific to the field of physical activity. The ranking will be different in other policy areas, with international organizations sometimes even surpassing that of national governments (e.g. WTO trade regulations or EU customs, monetary, fisheries or commercial policy).

Eventually, these considerations yielded a nine-field typology that distinguishes between documents relating to the three different categories of evidence on physical activity (x-axis) and ranging from low to medium and high degrees of coercion (y-axis).

In a second step, we used a limited set of eleven physical activity-related documents issued by scientific institutions, NGOs, national government institutions, and international organizations to conduct a preliminary test of the functions, characteristics, and potential utility of the matrix. For this exploratory exercise, we purposively sampled documents that we considered sufficiently relevant, recent, typical for certain aspects of the field of physical activity promotion, and illustrative of the problem of unclear document monikers. We then placed each document into the most appropriate field of the matrix based on its contents (x-axis/category) and the political influence exerted by its authoring institution (y-axis/degree of compulsion). Where necessary, documents were placed on the borders between types.

## Results

**Fig. 1:**
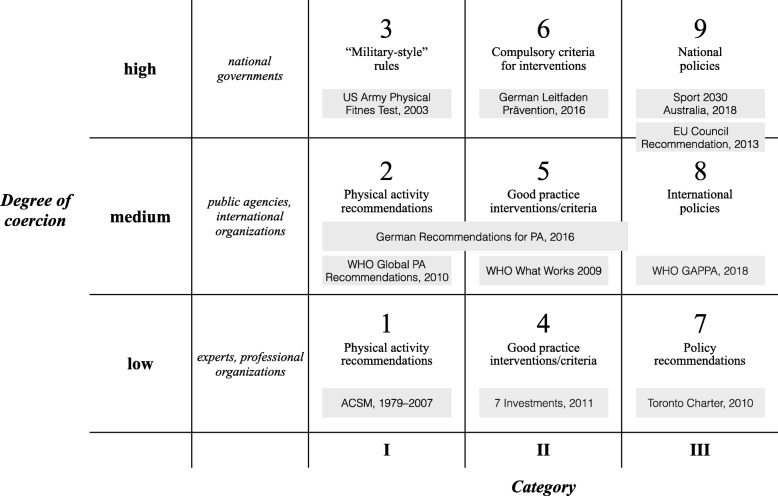
Typology and examples of documents on physical activity promotion

Figure 1 presents an overview of the typology and of the examples for the position of select documents within the matrix. The following sections provide short descriptions of the characteristics of each field, its relation and delineation from other fields, and the example used to illustrate it.

### Documents related to individual physical activity and health

The first column of the typology contains documents that are related directly to individual physical activity, and that are usually based on evidence that links physical activity to health outcomes [7]. *Field 1* would consist of the “classic” recommendations based on expert consensus about how much physical activity individuals should engage in to maintain or improve their health. One well known example are the “150 minutes of moderate-intensity exercise per week” [16], published as “guidelines”, “guidance”, “position statement” or “recommendation” by the American College of Sports Medicine (ACSM) [1, 16–19]. Statements of ACSM have usually been highly influential for the subsequent development of the field on a global scale, but they have never been binding for American citizens, doctors or administrations at the local, state or federal level.

A prominent example for *Field 2* are the WHO Global Recommendations on Physical Activity for Health [5]. Like ACSM, this document recommends at least 150 min of moderate to vigorous physical activity per week for healthy adults, with additional recommendations for children, adolescents and older people. Documents of *Field 2* have the same perspective and goals as recommendations placed in *Field 1*, but we argue that the fact that they were published or officially endorsed by a national or international public organization gives them a higher degree of political clout.

For physical activity, *Field 3* constitutes an almost “hypothetical” category, as there are much fewer laws and regulations – unlike, for example, in the areas of tobacco control (smoking bans), alcohol use (restriction of sale to minors). Documents formally regulating individual physical activity do exist, but only in closed environments: Public organizations such as armies, police forces and fire departments prescribe fitness levels or specific regular physical activities for their forces and enforce them through various means. For example, the US Army requires all its active soldiers to take the Army Physical Fitness Test twice a year and to score a minimum of 180 out of 300 points; failing the test consecutively may lead to exclusion from the force [20].

### Documents related to physical activity interventions

The next three boxes of the matrix are not directly related to the question of optimal individual physical activity levels but to collective action to promote physical activity, i.e. to “evidence that links interventions to physical activity behavior” [7] (category II). This evidence comes from the large body of research conducted on the effectiveness of different types of physical activity interventions. The goal of documents in the second column of the typology is to recommend (with varying degrees of coercion) “good” or “best practice” interventions to increase physical activity levels in the population.

*Field 4* might contain documents originating from scientific projects that select suitable interventions for the general population or specific age and target groups and showcase them to policy-makers and professionals in order to provide inspiration for implementation in additional places or settings. These documents have in common that they do not come from public institutions and therefore have a low degree of coercion; instead, they act as “a source of inspiration, learning and practical guidance” for practitioners and health promotion organizations [21]. As an example, “Investments that Work for Physical Activity” [22] (sometimes also referred to as “The 7 Investments” for short) highlights good practice interventions in schools, transport, urban design, health care, public education, community and sport for all. It accompanies the Toronto Charter [23] (see *Field 7* below) and was published by the Global Advocacy Council of the International Society for Physical Activity and Health (ISPAH) – a membership organization for researchers, practitioners, and policy-makers.

Recommendations by national governments or international organizations on interventions to promote physical activity in different sectors or for different age and target groups would fall into *Field 5*. These documents arguably have a higher degree of coercion as they come from “official” organizations with a considerable reputation and high level of visibility. However, they are non-coercive in that they do not “force” physical activity promoters to utilize the recommended interventions. A prominent example is the WHO report “Interventions on Diet and Physical Activity: What works” [24], which systematically reviewed the available scientific evidence for interventions in eight different categories and made recommendations based on a quality ranking.

*Field 6* pertains to “recommendations” for physical activity interventions that have a highly compulsory character, i.e. that are actually enforceable. Theoretically, governments (esp. at the national level) could prescribe certain interventions to organizations active in physical activity promotion, similar to regulations on food labeling or formulation in the area of nutrition or on advertising for tobacco and alcohol. They tend not to do this, but again, examples for such a mechanism exist in more limited environments (as in field 3). The “Leitfaden Prävention” (Prevention Guideline) of the German Health Insurance Association stipulates a set of criteria that interventions must meet in order to become eligible for reimbursement by the semi-public German sickness funds, i.e. sickness funds may only conduct or subsidize interventions that meet these criteria. Independent providers have to prove the eligibility of their offers to a central certification body. Insured persons engaging in approved courses can submit a certificate of participation to their sickness fund and claim partial or full reimbursement for their program fees [25].

### Documents related to physical activity policies

The third column of the matrix (*Fields 7, 8 and 9*) covers physical activity policy. It is based on research evidence for effective physical activity policies (category III). “Policy” has been defined as “legislative or regulatory action taken by federal, state, city, or local governments, government agencies, or non-governmental organizations such as schools or corporations” [26]. Research into effective policies could, for example, try to assess whether intersectoral government action is more effective in increasing physical activity levels than actions directed by a single sector. As with the other two categories of evidence, there is a continuum between recommendations with a low degree of coercion, usually published by experts or advocacy organizations, and highly binding documents, usually national policies. For the purposes of this typology, we suggest to limit our scope to public/government policy. One reason is that public policy constitutes the bulk of this category; another is that organizational policies (e.g. school policies) often tend to be confounded with interventions and are often reported as part of category II evidence [7].

A suitable example for *Field 7* is the Toronto Charter [23]. Published alongside “Investments that Work for Physical Activity” (see *Field 4*), the Charter is the more general, strategic document of the two. It argues for the need to address four key areas: national policies and action plans, policies that support physical activity, funding for physical activity and a corresponding reorientation of services, and partnerships for action. As it was authored by the Global Advocacy Council of ISPAH, the Charter has a relatively low degree of coercion and is not politically binding in any way.

Some WHO Strategies seem to fall into *Field 8* of the typology. A prominent and recent example is the Global Action Plan on Physical Activity [3]. On the one hand, such documents are much more “compulsory” than any policy recommendation issued by expert or advocacy groups. They are formally adopted by Member States, and all signatories commit to their implementation to a certain degree. However, these documents do not stipulate any specific compulsory goals to be reached by Member States, nor specific measures to be taken. Instead, they mostly “suggest” possible courses of action that countries may choose to either adopt, adapt or choose not to implement based to their specific national context.

*Field 9* would typically be populated by national physical activity promotion documents such as the “Sport 2030” plan by the Australian Federal government [27]. This recent policy stipulates that the government will introduce programs to reduce barriers to participation in physical activity, fund sport organizations and other partners to promote physical activity, and coordinate activities with sub-national governments and non-governmental organizations. Since it is officially published by the government itself, such a document has a much more politically binding character for national policy-making than the documents in fields 7 and 8, which a country can either opt out of or even disregard altogether.

### Documents on the boundaries: horizontal or vertical overlaps

Two final examples show that some documents do not clearly fall into one of the nine types but ‘sit on the boundaries’ between them or may partially ‘reach’ from one field into another horizontally (i.e. between categories/columns) or vertically (i.e. between degrees of coercion/lines).

A typical example for a horizontal overlap are the German National Recommendations for Physical Activity and Physical Activity Promotion [28], which may be filed into *Fields 2, 5, and 8* of the typology. Developed by a team of experts but officially tendered, endorsed and published by the Federal Ministry of Health, one can consider the document to have a medium degree of coercion. But as the name suggests, it covers both category I and II (i.e. recommendations for individuals and for appropriate interventions), and even extends into category III in several places by suggesting specific policy action (including intersectoral coordination, transport regulation, and fiscal incentive mechanisms).

A document situated vertically between the different degrees of coercion is the European Council Recommendation on Health-Enhancing Physical Activity across Sectors [4]. It is a policy document that exerts less political influence than a national policy (*Field 9*) but far more than typical documents from *Field 8*. The reason is that its originator, the EU, is a *supra*-national organization with far greater leverage on its member states than *inter*national organizations such as WHO [29, 30]. EU Member States will not face direct sanctions if they fail to implement the recommendation but may be exposed to peer pressure by other governments. In addition, the document obliges the European Commission to invest resources to support implementation and monitoring, increasing the chances for a potential impact of the document on national policy-making.

## Discussion

This article has put forth a typology of documents for physical activity promotion based on their degree of coercion and on the category of evidence they refer to. Our exploratory exercise with eleven documents shows that it may be used to structure the large number of available documents and provide a clearer view of their different purposes, target groups, and political clout. It reveals that documents with similar titles may be located in entirely different areas of the typology (e.g. the ACSM Recommendation [19] in *Field 1*, the German Recommendations for physical activity [28] in *Field 2/5*, or the EU Council Recommendation [4] in *Field 8/9*], that documents with very different names may be similar in nature (e.g. the Australia 2030 sport plan [27] and the EU Council Recommendation [4], both in *Field 9*), or that documents accompanying each other may have slightly different perspectives (e.g. The 7 Investments [22] in *Field 4* and the Toronto Charter [23] in *Field 7*).

We are aware that the typology has some limitations that need to be taken into account. Like other typologies in the field of Public Health, such as Frieden’s Health Impact Pyramid [31], it necessarily constitutes a simplification of reality and will not be able to accommodate all cases and variations. It is inherent in typologies that they can never be a full and precise depiction of reality and that it will always remain impossible to fit all cases neatly into specific fields. As the two examples mentioned above [4, 28] have shown, there will always be documents transcending boundaries between fields, both horizontally and vertically.

In general, alternative choices may have been possible for either of the two axes of the matrix. There are several other typologies that distinguish between types of evidence on health and/or physical activity, notably by Brownson et al. [32] and Martin-Diener et al. [33], but we opted for the one by Rütten et al. [7] as it allows us to link our considerations to the most recent overview of the literature. A more radical alternative would be the use of a socio-ecological or bio-psycho-social model [34–36], which provides a more detailed range of categories from individual via social-interactive to environmental and political factors. However, we ended up opting for the proposed tripartite distinction as (a) many policy documents address multiple categories within the socio-ecological model, making it difficult to locate them properly, and (b) we felt that a parsimonious solution would be most appropriate to present our general argument. Likewise, the literature on policy instruments provides alternatives to the Doern continuum, but many of these include a large variety of categories [e.g. 9] that do not form a continuum or that already have multiple dimensions [e.g. 8, 11], which would have added too much complexity to our own matrix.

These arguments notwithstanding, our choice of dimensions may also spark criticism: Regarding the x-axis, it may be empirically difficult to distinguish between the different categories of evidence, particularly between *Categories II* (interventions) *and III* (policy). In their scoping review, Rütten and colleagues acknowledge that making this distinction is an idea that has not yet been widely picked up in the field, and that even research publications often conflate interventions and policy for physical activity promotion [7]. This may render it particularly difficult to place documents into one of these two categories. On the y-axis, the Doern continuum has been criticized as being difficult to operationalize in practice [8] and, as the authors themselves have conceded, for arranging policies “somewhat artificially” [13]. However, as Howlett observes, the idea remains extremely popular in the field of policy studies, “its virtues of simplicity and parsimony apparently outweighing its empirical and conceptual difficulties” [8].

Some aspects implied by the typology may require further empirical testing. For example, distinguishing between documents in *Fields 1 and 2* hinges on the hypothesis that individuals (and/or practitioners) will be influenced more by physical activity recommendations made by governmental institutions than by experts – but this may not be the case, or even the opposite might be true if levels of distrust in the government are high. Further research would be needed to test whether the assumed higher degree of coercion of governmental documents actually exists in *Category I*, and similar empirical studies could be conceived for the other two categories (i.e. *Field 4* vs. *5* and *Field 7* vs. *8*).

We have used a small set of particularly illustrative publications to explore the utility of the matrix, but a number of other potentially relevant documents immediately come to mind. This includes the Physical Activity Guidelines for Americans [37] (potentially suitable for *Field 2*), the CDC Guide to Strategies to Increase Physical Activity in the Community [38] (possibly *Field 5*), the Physical Activity Strategy for the WHO European Region 2016–2025 [6] (potentially *Field 8*), or national policies such as Get Ireland Active [39] and Moving More, Living More from the UK [40] (possibly *Field 9*). We believe our results show that fully populating the matrix would be a worthwhile endeavor; doing so would require conducting a systematic search for eligible documents and assigning them to the fields of the typology in a structured fashion based on a further operationalization of the underlying theoretical concepts.

Another interesting exercise may be to examine whether a similar diversity of document monikers exists in other fields of health promotion – particularly to the other “big” areas in the prevention of non-communicable diseases, i.e. tobacco, alcohol and nutrition – and whether our proposed typology could also be useful there. It might also be stimulating to compare the degree of coercion of documents across domains, e.g. whether the political clout of publications by international organizations is potentially higher in other policy areas (e.g. the role of the EU in the field of food regulation).

## Conclusions

We believe that the typology proposed in this article will improve practitioners’ and policy-makers’ understanding of the multitude of existing documents for physical activity promotion. It may help them look beyond the titles of documents and more specifically for the categories of evidence that they are interested in, and it may direct them to documents at the appropriate political level that could serve as blueprints for their own planned activities. In addition, it may spark further conceptual and empirical research and even inspire scholars and experts from other areas of health promotion. The proposed typology has its caveats, but it fills an important gap in health promotion theory development, as we are not aware of any other framework in the current literature that would address this issue. Thus, the typology in its current form may serve as a point of departure for further discussion, refinement, and modifications.

Placing further documents in the typology may help us identify important gaps for future action. At the national level, this could guide the future development of policy documents, based on what is already available nationally and what is not; at the international level, it may spark discussions about both future research and about the general direction of physical activity guidance and policy. For example, do we need more binding policy at the individual level (e.g. laws and taxation in *Field 3* rather than recommendations in *Fields 1 and 2*) and more regulation on physical activity interventions (e.g. binding funding criteria in *Field 6* rather than good practice collections in *Fields 4 and 5*)? Or would it be wise not to over-regulate physical activity and maintain the current approach? Such questions are potentially relevant to both researchers, practitioners, and policy-makers in physical activity promotion, but they may not even be asked if we do not have a proper overview of the lay of the land.

## Data Availability

Not applicable.

## Abbreviations

ACSM: American College of Sports Medicine
CDC: Centers for Disease Control and Prevention
EU: European Union
ISPAH: International Society for Physical Activity and Health
WHO: World Health Organization
WTO: World Trade Organization
